# Exenatide inhibits NF-κB and attenuates ER stress in diabetic cardiomyocyte models

**DOI:** 10.18632/aging.103181

**Published:** 2020-05-11

**Authors:** Zhenhong Fu, David Mui, Hang Zhu, Ying Zhang

**Affiliations:** 1 Department of Cardiology, The First Medical Center, Chinese PLA General Hospital, Beijing, China; 2 Perelman School of Medicine, University of Pennsylvania, Philadelphia, PA 19104, USA

**Keywords:** ER stress, exenatide, hyperglycemia, NF-κB signaling pathway, cardiomyocyte

## Abstract

Exenatide is used to treat patients with type-2 diabetes and it also exerts cardioprotective effects. Here, we tested whether Exenatide attenuates hyperglycemia-related cardiomyocyte damage by inhibiting endoplasmic reticulum (ER) stress and the NF-κB signaling pathway. Our results demonstrated that hyperglycemia activates the NF-κB signaling pathway, eliciting ER stress. We also observed cardiomyocyte contractile dysfunction, inflammation, and cell apoptosis induced by hyperglycemia. Exenatide treatment inhibited inflammation, improved cardiomyocyte contractile function, and rescued cardiomyocyte viability. Notably, re-activation of the NF-κB signaling pathway abolished Exenatide’s protective effects on hyperglycemic cardiomyocytes. Taken together, our results demonstrate that Exenatide directly reduces hyperglycemia-induced cardiomyocyte damage by inhibiting ER stress and inactivating the NF-κB signaling pathway.

## INTRODUCTION

Excessive glucose intake is considered an independent risk factor for the development of obesity and diabetes. The primary complications of diabetes or obesity include diabetic cardiomyopathy, neuropathy, hyperglycemia-related kidney damage, retinopathy, and food damage. Among these, cardiovascular complications account for most of diabetes-related deaths [1]. Despite numerous efforts into advancing therapeutic approaches for diabetes, anti-diabetic medications do not seem to prevent the cardiovascular damage induced by diabetes. Recently, Exenatide, a glucagon-like protein-1 receptor agonist, was clinically used to treat patients with type-2 diabetes. While cardioprotective effects have been reported for Exenatide, the underlying molecular mechanisms remain unclear [2, 3].

Protein peroxidation promotes diabetes [4, 5]. Glycosylative, phosphorylative, and oxidative modifications of cytoplasmic proteins, induced by chronic hyperglycemia, change the normal patterns of protein folding and degradation in cells [6–8]. Additionally, abnormal post-transcriptional modifications correlate with disturbed glucose metabolism. In the cytoplasm, the endoplasmic reticulum (ER) is the main location for protein synthesis, modification, transport and release [9, 10]. Abnormal protein modifications seem to occur in the ER, followed by accumulation of unfolded proteins in the ER lumen. This process is termed “ER stress” and is regulated by PERK, ATF6 and IRE1 [11]. Although ER stress contributes to the development of diabetic cardiomyopathy [12, 13], there is no data explaining Exenatide’s effects on ER stress under hyperglycemia stress.

ER stress contributes to cellular inflammation and death through distinct signaling pathways [14, 15]. NF-κB promotes inflammation by upregulating the transcription of pro-inflammatory factors [16]. In addition, NF-κB may promote ER stress by upregulating the transcription and activity of proteins in the JNK pathway [17]. Furthermore, NF-κB is also a downstream effector of hyperglycemia and increased NF-κB activity has been noted in hyperglycemia-treated cardiomyocytes [18, 19]. Therefore, this suggests that NF-κB may promote ER stress induced by hyperglycemia. Accordingly, here we tested the hypothesis that Exenatide attenuates hyperglycemia-related cardiomyocyte damage by inhibiting ER stress and the NF-κB signaling pathway.

## RESULTS

### Exenatide improves cardiomyocyte viability and reduces inflammation response induced by hyperglycemia

**Figure 1:**
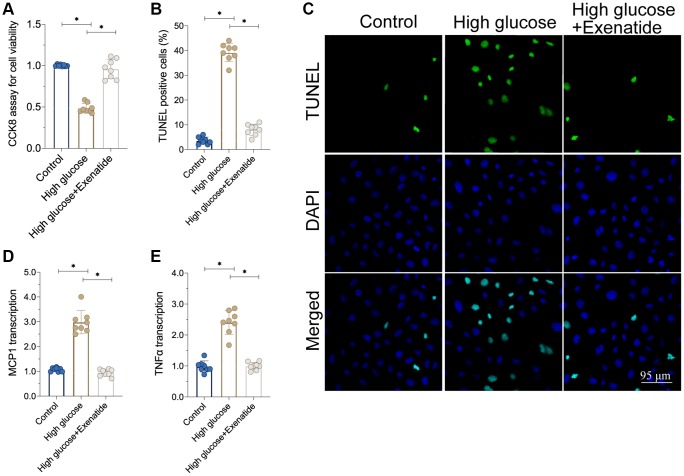
**Exenatide attenuates hyperglycemia-induced cell apoptosis and inflammation.** (**A**) CCK8 assay for cell viability. (**B**, **C**) TUNEL staining for apoptotic cells. (**D**, **E**) qPCR assay for MCP1 and TNFα transcription. *P<0.05.

In this study, hyperglycemia was induced in cardiomyocytes [20], which were then treated with Exenatide. The viability of cardiomyocytes was determined through CCK8 assay. As shown in Figure 1A, cardiomyocyte viability was reduced in response to hyperglycemia whereas exenatide treatment improved it. In agreement with this effect, we also observed an increase in TUNEL-stained apoptotic cells after hyperglycemia treatment (Figure 1B, 1C). Exenatide treatment reduced the ratio of TUNEL-positive cells, reconfirming that such treatment increases cardiomyocyte viability and survival. In addition to cell death, we also assessed the inflammation response by measuring the levels of pro-inflammatory factors. As shown in Figure 1D, 1E, compared to the control group, MCP1 and TNFα transcription was elevated in hyperglycemic cells. On the other hand, Exenatide treatment inhibited the upregulation of such pro-inflammatory factors, suggesting that Exenatide inhibits hyperglycemia-induced inflammation in cardiomyocytes.

### Exenatide treatment enhances the function of hyperglycemia-treated cardiomyocytes

**Figure 2:**
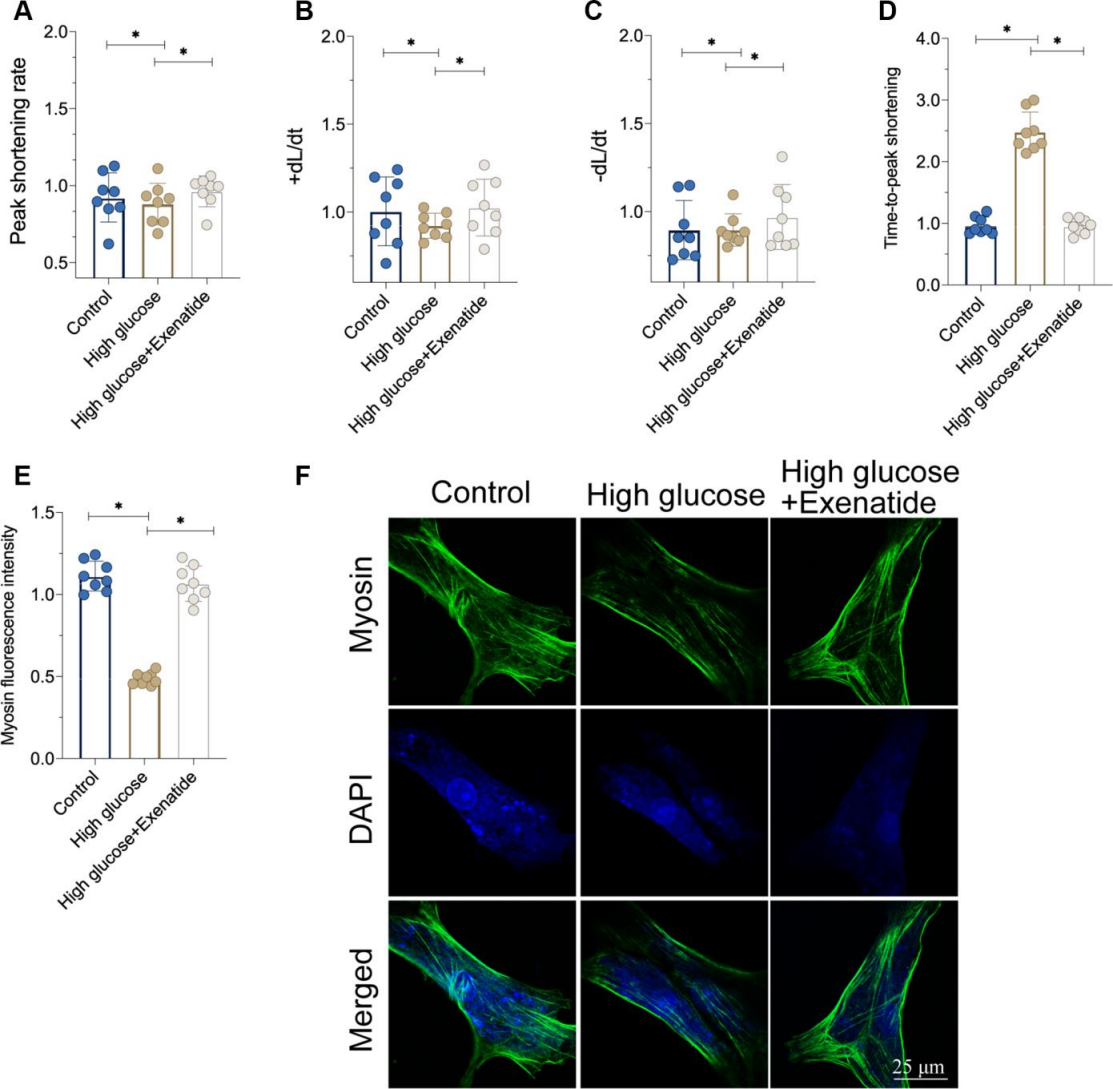
**Exenatide treatment improves cardiomyocytes function under hyperglycemia stress.** (**A**–**D**) Signal of cardiomyocyte contractile parameters measured in response to Exenatide treatment. (**E**, **F**) Myosin expression determined through immunofluorescence. *P<0.05.

We isolated single cardiomyocytes after hyperglycemia treatment and assessed their contractile properties [21, 22]. As shown in Figure 2A–2D, the peak shortening rate was downregulated in the hyperglycemia group. Similarly, the maximal velocity of shortening (+dL/dt) and maximal velocity of re-lengthening (−dL/dt) were also impaired by hyperglycemia in cardiomyocytes. The time-to-peak shortening (TPS) was elevated in hyperglycemic cardiomyocytes when compared to controls. Therefore, this indicates that cardiomyocytes contraction and relaxation functions are compromised by hyperglycemia, whereas Exenatide treatment restored the peak shortening rate, +dL/dt, −dL/dt and TPS (Figure 2A–2D). This suggests that cardiomyocyte function could be sustained by Exenatide under hyperglycemic stress. At the molecular level, cardiomyocyte contractility is controlled by cytoskeletal proteins such as Myosin. Interestingly, Myosin levels were reduced in hyperglycemic cardiomyocytes whereas Exenatide treatment increased them, thereby rescuing contractile functions.

### Exenatide inhibits ER stress and the NF-κB signaling pathway

**Figure 3:**
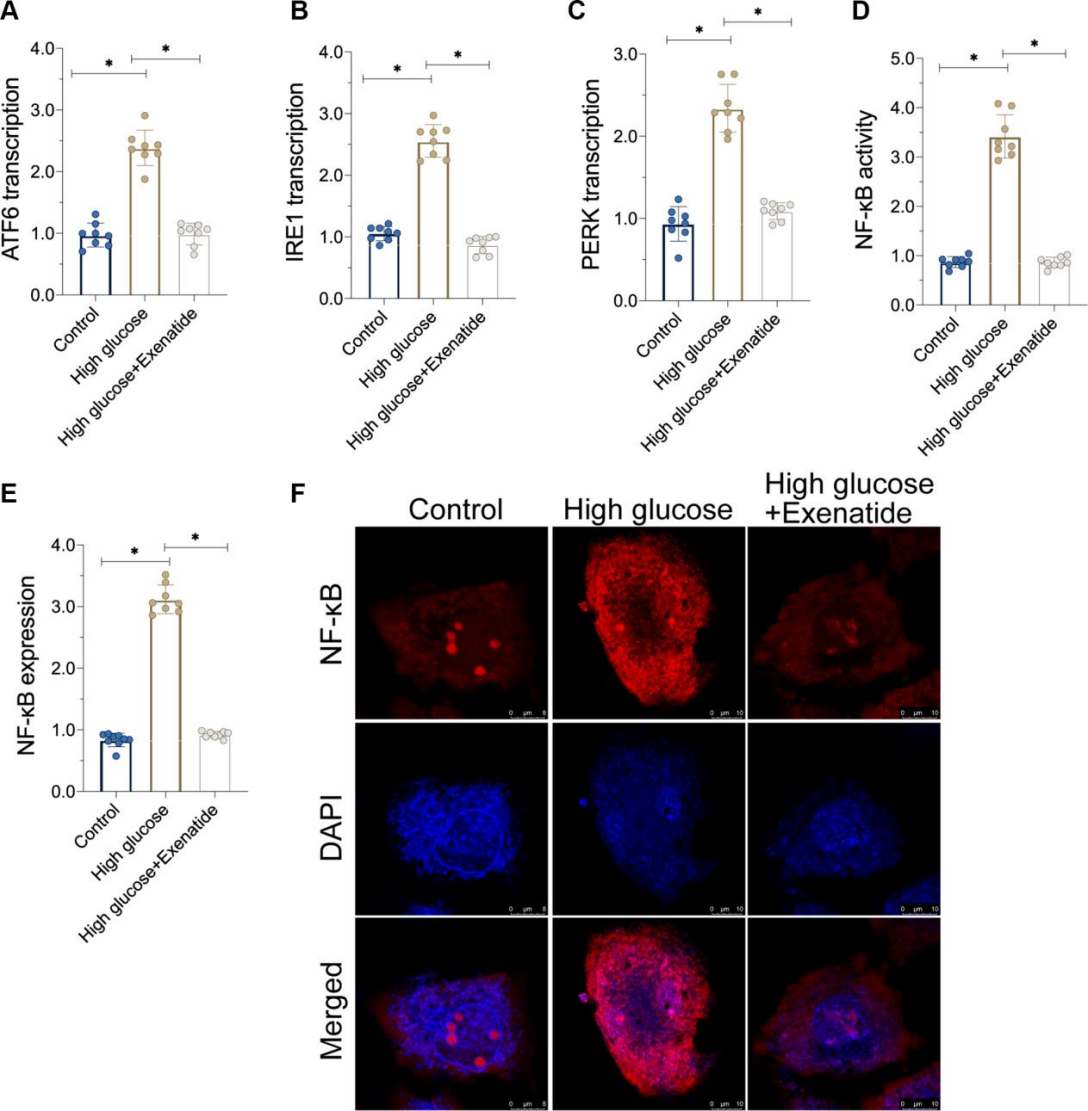
**Exenatide reduces ER stress and the activation of NF-κB signaling pathway.** (**A**–**C**) qPCR assay for ATF6, IRE1 and PERK transcription. (**D**) ELISA assay for NF-κB activity. (**E**–**F**) Immunofluorescence staining for NF-κB. *P<0.05.

As discussed above, hyperglycemia-induced cardiomyocyte damage is associated with ER stress and NF-κB signaling pathway activation [23, 24]. Accordingly, we observed alterations in ER stress and NF-κB activation in response to Exenatide treatment. As shown in Figure 3A–3C, the transcriptions of PERK, ATF6 and IRE1 were increased in response to hyperglycemia treatment, suggesting an activation of ER stress. In addition, we also found that the activity of NF-κB was also augmented in hyperglycemic cells (Figure 3D). Interestingly, Exenatide treatment reduced the levels of PERK, ATF6 and IRE1 (Figure 3A–3C), suggesting that Exenatide inhibits ER stress. In addition, NF-κB activity was also inhibited by Exenatide treatment (Figure 3D). We also observed an upregulation in NF-κB expression in hyperglycemic cells through immunofluorescence assays while Exenatide treatment reduced NF-κB expression to near-normal levels (Figure 3E, 3F). Overall, our results indicate that Exenatide regulates inhibits ER stress and the NF-κB signaling pathway.

### Re-activation of the NF-κB pathway abolishes Exenatide-mediated ER stress inhibition

**Figure 4:**
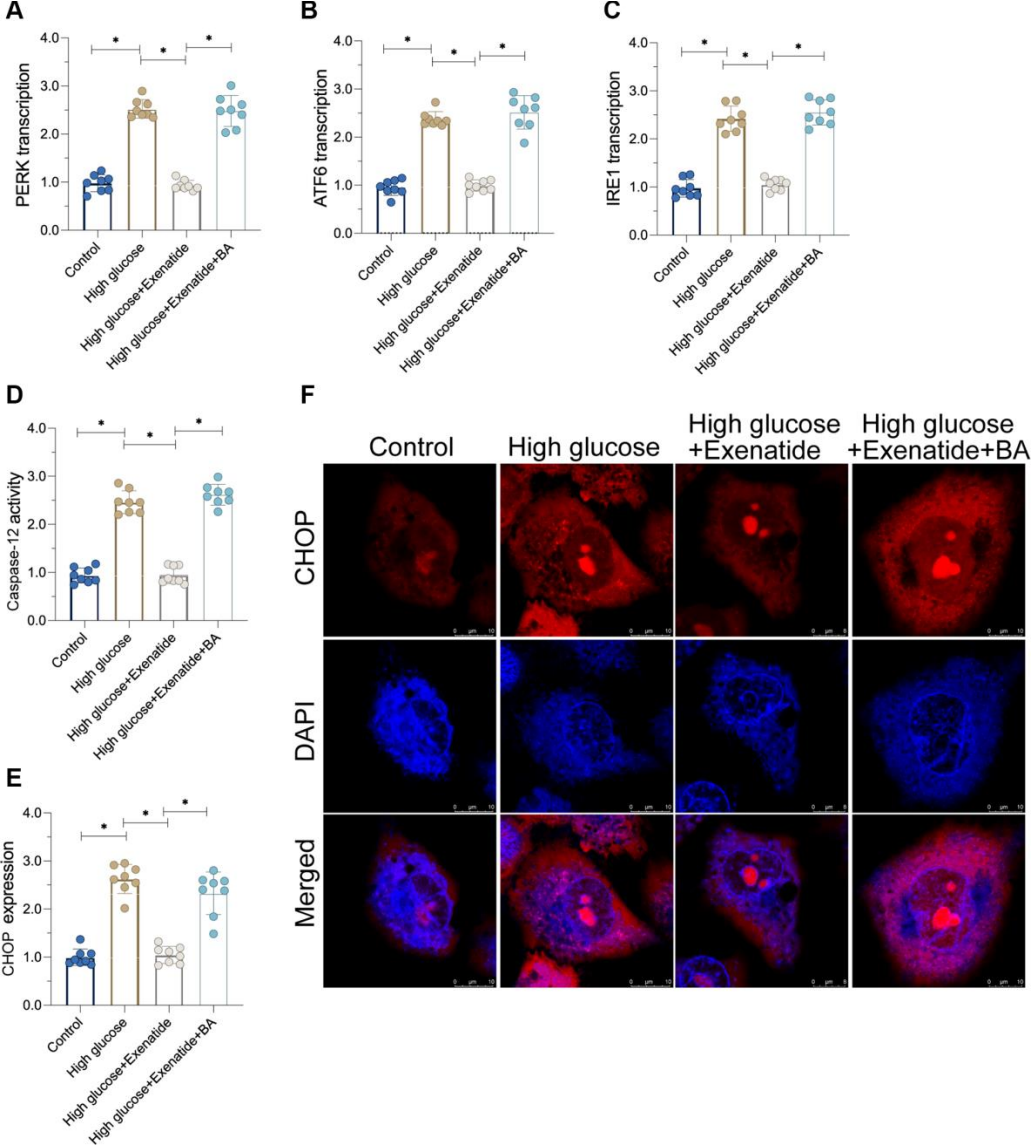
**Re-activation of NF-κB promotes cardiomyocyte damage.** (**A**–**C**) qPCR assay for ATF6, IRE1 and PERK transcription in the presence of BA to activate NF-κB. (**D**) ELISA assay for caspase-12 activity. (**E**, **F**) Immunofluorescence staining for CHOP. *P<0.05.

To verify whether NF-κB pathway is required for Exenatide-induced ER stress protection [25], we added an agonist, Betulinic acid (BA), to activate the NF-κB pathway. Then, ER stress was analyzed again. As shown in Figure 4A–4C, ER stress markers were elevated in the hyperglycemia group compared to controls. Exenatide treatment prevented the upregulation of ER stress-related markers whereas these effects were not evident in BA-treated cardiomyocytes. Thus, our results indicate that inhibition of the NF-κB pathway by Exenatide is required for ER stress inhibition. Additionally, we also measured the activity of caspase-12 and CHOP. As shown in Figure 4D–4F, we observed increased caspase-12 and CHOP activity in the hyperglycemia group. Although Exenatide treatment inhibited caspase-12 and CHOP activity, this action is not apparent in BA-treated cardiomyocytes. Altogether, our results confirm that Exenatide inhibits ER stress through the NF-κB pathway.

### Re-activation of the NF-κB pathway promotes cardiomyocyte inflammation and death

**Figure 5:**
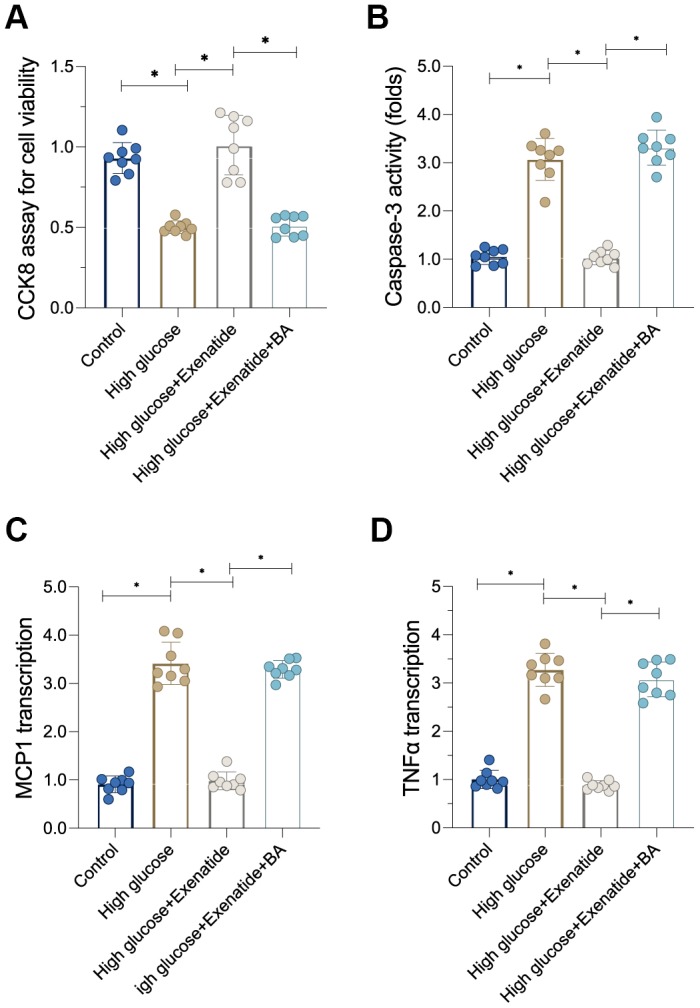
**Re-activation of NF-κB induces cardiomyocyte death and inflammation.** (**A**) CCK8 assay for cell viability. (**B**) ELISA assay for caspase-3 activity. (**C**, **D**) qPCR assay for MCP1 and TNFα transcription. *P<0.05.

Next, we sought to determine whether the NF-κB pathway is involved in Exenatide-regulated cardiomyocyte death and inflammation [26]. First, cardiomyocyte viability, as evaluated through CCK8 assay, was reduced after exposure to hyperglycemia treatment whereas Exenatide treatment restored cardiomyocyte viability in a NF-κB-dependent manner. Indeed, re-activation of the NF-κB pathway abolished the pro-survival effects of Exenatide on cardiomyocytes (Figure 5A). In addition to CCK8 assay, we also measured the activity of caspase-3, a critical regulator of cell apoptosis. As shown in Figure 5B, caspase-3 activity was increased in the hyperglycemia group. Exenatide caused a decrease in caspase-3 activity whereas re-activation of the NF-κB pathway through supplementation with BA induced an activation of caspase-3 in Exenatide-treated cardiomyocytes. Overall, our results verified that NF-κB pathway inhibition accounts for Exenatide-mediated cardiomyocyte survival. In addition to cardiomyocyte viability, we also evaluated the inflammation response. Pro-inflammatory factors were upregulated in response to hyperglycemia treatment whereas Exenatide treatment inhibited the transcription of inflammatory factors (Figure 5C, 5D). Re-activation of the NF-κB pathway abolished Exenatide-mediated inflammation inhibition. Therefore, our data suggest that Exenatide also promotes cardiomyocyte viability and inhibits inflammation through the NF-κB pathway.

## DISCUSSION

Diabetic cardiomyopathy is caused by chronic hyperglycemia stress. Cardiomyocyte apoptosis and microvascular damage are the primary factors promoting the development of diabetic cardiomyopathy [27]. The clinical features of diabetic cardiomyopathy include decreased cardiac output and limited cardiac relaxation. Additionally, cardiovascular disorders are the most dangerous complication for diabetic patients [28, 29]. More than 40% of patients with type-2 diabetes will suffer from cardiovascular complications and more than half of those will die from diabetic cardiomyopathy. Although insulin administration can treat type-2 diabetes, its chronic use increases the risk of cancer. Furthermore, most patients often present hypoglycemia after insulin treatment, with no beneficial effects on diabetes-related cardiovascular complications.

Exenatide is a recently developed drug to treat diabetes [30, 31]. The molecular mechanisms underlying Exenatide’s functions involve GLP-1 receptor activation, which promotes glucose utilization. Exenatide elicits only a low hypoglycemic response and cardiovascular benefits increase with extended use of the drug [32]. In this study, we investigated the mechanisms underlying Exenatide-mediated cardio-protection *in vitro*.

ER stress entails a series of protein modification and folding/unfolding events in response to abnormal quantities and decreased quality of proteins in the ER lumen [13, 33]. Although proteins are primarily translated from mRNA by ribosomes, the functional configurations and post-transcriptional modifications of many proteins are attained in the ER [34, 35]. After chronic hyperglycemia, the levels of glucose in the cytoplasm are high, contributing to protein degradation and oxidation. Therefore, ER stress is followed by abnormal protein accumulation [36, 37]. Our data here indicated that hyperglycemia treatment triggered ER stress, followed by cardiomyocyte dysfunction and death. Interestingly, Exenatide treatment attenuated ER stress, thereby sustaining cardiomyocyte contractile functions and favoring cardiomyocyte survival [38, 39]. These results identified ER stress as a downstream target of Exenatide treatment. Indeed, protection of the ER against abnormal protein modifications promotes cardiomyocyte viability [40, 41].

In our study, we found that inflammation and ER stress were under the control of the NF-κB pathway. In contrast, hyperglycemia-activated NF-κB was inhibited by Exenatide. These results demonstrate that Exenatide exerts protective effects on cardiomyocytes through the NF-κB signaling pathway, in agreement with previous studies [42, 43]. Indeed, such studies have shown that inhibition of NF-κB prevents heart inflammation [44, 45] while improving oxidative stress and countering the downregulation of anti-oxidative factors [46, 47]. In addition, myocardial damage induced by a high-fat diet was also normalized by NF-κB inhibition [48]. The pro-inflammatory role of NF-κB in cardiomyocyte damage has also been observed in myocardial ischemia-reperfusion injury [49, 50].

While our observations will need to be validated in clinical settings, our results here demonstrated that Exenatide treatment inhibited inflammation and oxidative stress and improved viability in cardiomyocytes during hyperglycemia stress. Specifically, Exenatide inhibited the NF-κB pathway and attenuated ER stress, supporting cardiomyocyte survival. These findings highlight NF-κB as a potential therapeutic target in the treatment of diabetes-induced cardiomyopathies.

## MATERIALS AND METHODS

### Cell culture

We cultured H9C2 cells, as previously described [51]. Hyperglycemia stimulation was mimicked as previously reported [52]. In brief, H9C2 cells were cultivated in DMEM medium supplemented with EGF Single Quots (PELOBiotech GmbH, Martinsried, Germany) plus 10% FBS and used until passage five. Hyperglycemia stress was induced by incubating the cells in 25 mmol/L high glucose medium for 12 h. The cells were treated with exenatide (10 μM) and incubated for 12 h before high-glucose treatment. Betulinic acid (BA) at 5 nM was added to activate the NF-κB signaling pathway [53].

### Immunoblotting

Proteins were extracted from cell lysates (McA or primary mouse hepatocytes) and tissue lysates (liver) in RIPA buffer (50 mM Tris pH 7.4, 150 mM NaCl, 0.25% sodium deoxycholate, 1% Nonidet P-40) [54]. Total protein amounts were quantified using Bio-Rad DC assay kit (Bio-Rad, Hercules, CA). In general, 20-80 μg of protein homogenate were separated by SDS-PAGE and subsequently electroblotted onto PVDF membranes (Bio-Rad). Membranes were blocked with fat-free milk powder (5% w/v) dissolved in Tris-buffered saline (15 mM NaCl and 10 mM Tris/HCl, pH 7.5) containing 0.01% Tween 100 (TBS-T), washed, and incubated overnight at 4 °C with the appropriate primary antibody (see above) [55]. Infrared fluorescent-labeled secondary antibodies were prepared at 1:15,000 dilution in TBS-T with 5% fat-free milk powder and incubated for 1 h at room temperature. Protein band quantification was performed using ImageJ (NIH, Bethesda, MD) [56].

### Immunofluorescence and confocal microscopy

Cells were grown on fibronectin-coated glass coverslips. After administering the corresponding treatments, the media were removed and cell monolayers were washed with ice-cold PBS. The cells were then fixed with 7.5% of neutral-buffered formalin solution containing 0.025% of glutaraldehyde for 10 min at room temperature [57]. The specimens were then washed and permeabilized with PBS containing 0.1% Triton (PBS-T) or 0.1% digitonin, and blocked for 1 h with PBS supplemented with 5% bovine serum albumin and 2% goat serum (Sigma) [58]. Afterwards, specimens were incubated overnight in blocking buffer containing the corresponding primary antibodies at a 1:200 concentration. Specimens were washed and incubated for 1 h at room temperature with the corresponding secondary antibodies (see above) at a 1:400 dilution in blocking buffer. Secondary antibodies conjugated to Alexa fluorophores were purchased from Molecular Probes (Life Technologies). DAPI was used to stain nuclei [59]. For some co-localization experiments, when two primary antibodies were raised in rabbits, one of the primary antibodies was detected using the regular procedure, while the second primary antibody was conjugated with Alexa fluorophore 488 using a commercially available kit (Life Technologies) [60]. Confocal images were acquired with a Leica TCS SP5 II confocal microscope by using a 405 diode laser (excitation 405 nm), a multiline argon laser (excitation 488 nm), and two HeNe lasers (excitation 543 and 633 nm) with a 40× Apochromat, numerical aperture 1.25 – Oil objective and with a 63× Apochromat, numerical aperture 1.40- Oil objective [1].

### RNA isolation and Quantitative Real Time (QRT)-PCR analysis

RNA was isolated from cultured cells using TRIzol Reagent following the manufacturer’s instructions (Life Technologies) [61, 62]. Two μg of total RNA were reverse-transcribed to cDNA using the Verso cDNA Synthesis kit (ThermoFisher Scientific). QRT-PCR was carried out with TaqMan chemistry and probes (Applied Biosystems). Gene expression analyses were performed with the ABI Step One Plus QRT-PCR machine (Applied Biosystems) [63].

### Mitochondrial membrane potential assay

Mitochondrial membrane potential in cells was measured by JC-1 fluorescence intensity. In brief, cells were plated at 50,000 cells/well in a 96-well black bottom dish. After hyperglycemia stimulation, cells were incubated with 10 μM JC-1 and DAPI for 30 min in the dark [64]. Then, JC-1 and Hoechst (360/450) fluorescence intensity was measured using a SpectraMax3 plate reader (Molecular devices, Sunnyvale, California). The JC-1/DAPI fluorescence ratio is used to determine the mitochondrial membrane potential [65].

### Oxidative stress and superoxide production

Mitochondrial ROS production was measured with a fluorometric assay, as described [66]. Activity of aconitase was measured in isolated mitochondria using a spectrophotometric assay, and tissue ROS levels were measured by the conversion of nonfluorescent 2’,7’-dichlorofluorescein-diacetate (DCFDA) to the highly fluorescent 2’,7’-dichlorofluorescein (DCF), as described before. In cells, ROS levels were measured by CellRox reagent in accordance with the manufacturer’s instructions (Thermo Fisher Scientific, Waltham, MA) [67].

### Statistical analysis

All experiments were conducted in at least triplicate. Data are expressed as the mean ± SEM. Statistical differences were analyzed using GraphPad Prism software (GraphPad Software Inc., San Diego, CA). We tested for normality in the distribution of the sample groups using the D’Agostino-Pearson omnibus and the Shapiro-Wilk normality tests [68]. Statistical significance was then determined by unpaired two-tailed Student’s *t*-test with a threshold of significance set at *p*<0.05.
